# Genetic Susceptibility to Non-Necrotizing Erysipelas/Cellulitis

**DOI:** 10.1371/journal.pone.0056225

**Published:** 2013-02-20

**Authors:** Katariina Hannula-Jouppi, Satu Massinen, Tuula Siljander, Siru Mäkelä, Katja Kivinen, Rasko Leinonen, Hong Jiao, Päivi Aitos, Matti Karppelin, Jaana Vuopio, Jaana Syrjänen, Juha Kere

**Affiliations:** 1 Department of Medical Genetics, University of Helsinki, and Folkhälsan Institute of Genetics, University of Helsinki, Helsinki, Finland; 2 Department of Infectious Disease Surveillance and Control, National Institute for Health and Welfare, Helsinki, Finland; 3 Department of Biosciences and Nutrition, Center for Biosciences, Karolinska Institutet, Huddinge, Sweden; 4 Department of Internal Medicine, Tampere University Hospital, Tampere, Finland; 5 Medical School, University of Tampere, Tampere, Finland; 6 Science for Life Laboratory, Karolinska Institutet, Huddinge, Sweden; Cairo University, Egypt

## Abstract

**Background:**

Bacterial non-necrotizing erysipelas and cellulitis are often recurring, diffusely spreading infections of the skin and subcutaneous tissues caused most commonly by streptococci. Host genetic factors influence infection susceptibility but no extensive studies on the genetic determinants of human erysipelas exist.

**Methods:**

We performed genome-wide linkage with the 10,000 variant Human Mapping Array (HMA10K) array on 52 Finnish families with multiple erysipelas cases followed by microsatellite fine mapping of suggestive linkage peaks. A scan with the HMA250K array was subsequently performed with a subset of cases and controls.

**Results:**

Significant linkage was found at 9q34 (nonparametric multipoint linkage score (NPL_all_) 3.84, p = 0.026), which is syntenic to a quantitative trait locus for susceptibility to group A streptococci infections on chromosome 2 in mouse. Sequencing of candidate genes in the 9q34 region did not conclusively associate any to erysipelas/cellulitis susceptibility. Suggestive linkage (NPL_all_>3.0) was found at three loci: 3q22-24, 21q22, and 22q13. A subsequent denser genome scan with the HMA250K array supported the 3q22 locus, in which several SNPs in the promoter of *AGTR1* (Angiotensin II receptor type I) suggestively associated with erysipelas/cellulitis susceptibility.

**Conclusions:**

Specific host genetic factors may cause erysipelas/cellulitis susceptibility in humans.

## Introduction

Bacterial non-necrotizing erysipelas and cellulitis are often recurring, diffuse, and spreading infections of the skin and subcutaneous tissues, which manifest with local erythema, pain, and warmth usually accompanied by fever, leukocytosis, lymphangitis, and lymphadenitis [1]. Both Group A (*Streptococcus pyogenes*) and G (typically *Streptococcus dysgalactiae* subsp. *equisimilis*) β-hemolytic streptococci are the predominant causative agents of cellulitis/erysipelas but infections may also be caused by group B and C streptococci and *Staphylococcus aureus* [1], [2]. Risk factors for erysipelas/cellulitis include impaired lymphatic drainage, venous insufficiency, skin eruptions and trauma, and obesity [3]–[5]. Erysipelas/cellulitis causes significant morbidity and recurrence is common especially with tibial involvement, history of malignancy, dermatitis or prior surgery of the affected limb [3]–[6]. The course of a clinical infection is the outcome of the host genome, the pathogens’ virulence, and the environment. Interindividual variation between hosts can cause infections to range from asymptomatic to fatal infection, e.g. the same group A streptococcal (GAS) strain can be carried asymptomatically, cause uncomplicated pharyngitis or potentially fatal bacteremia such as streptococcal toxic shock syndrome or necrotizing fasciitis [7]. Twin, sibling, and adoption studies have recognized genetic factors as important determinants of susceptibility to infectious diseases, but recurrent infections and clustering in families still involve unknown genetic and immunological factors [8].

Mendelian and polygenic host genetic factors are known to influence susceptibility to infection by bacteria, parasites, and viruses including *Mycobacterium leprae*, *Mycobacterium tuberculosis*, *Streptococcus pneumoniae*, *Neisseria meningitidis*, *Schistosoma mansoni*, *Leishmania donovani*, Epstein-Barr virus, and human papilloma virus [9], [10]. Conversely, resistance is known for *Plasmodium vivax*, human immunodeficiency virus-1, meningococcal disease, and norovirus [9], [10]. Genome-wide scans have identified susceptibility loci for leishmaniasis (22q12, 2q23-31), leprosy (10p13, 6q25), and tuberculosis (15q, Xq) [11]–[14]. Host genetics is a significant factor in determining susceptibility to severe GAS sepsis in mouse and severe invasive GAS infection in humans [7], [15]. Mouse susceptibility loci for GAS infection have been mapped to chromosome 17, including the mouse MHC region (syntenic to human 6p21); chromosome 7, which is also linked with susceptibility to *Streptococcus pneumoniae* infection in mice (syntenic to human 19q13.1-13.3); and chromosome 2, including genes of the interleukin 1 alpha, and prostaglandin E synthetase pathways (syntenic to human 2q14 and 9q33-34) [16]–[18]. Specific Human Leukocyte Antigen class II (HLAII) haplotypes protect from severe systemic disease caused by GAS whereas other haplotypes increase the risk of severe disease [19], [20]. HLAII molecules are receptors for microbial superantigens and their allelic variations can regulate cytokine responses. The intensity of an individual’s inflammatory cytokine response correlates directly with the severity of infection: a higher cytokine response leads more often to severe systemic disease than lower cytokine levels [19]. The HLA/MHC region in humans has also been associated with the susceptibility to many other infectious diseases, e.g., HIV/AIDS, hepatitis, leprosy, tuberculosis, malaria, leishmaniasis, and schistosomiasis [21].

We have used erysipelas/cellulitis (hereafter referred to as erysipelas) as a marker infection to identify families with two or more family members suffering from erysipelas and suggesting a possibly increased susceptibility to streptococcal infections. To identify putative susceptibility loci we performed a whole-genome genetic linkage scan and identified suggestive loci on chromosomes 9q34, 3q22-24, 21q22, and 22q13.

## Materials and Methods

### Ethics Statement

This study was approved by the Ethical Review Board of Pirkanmaa Hospital District, Tampere, Finland. Written informed consent was obtained from all study participants. All clinical investigations have been conducted according to the principles expressed in the Declaration of Helsinki.

### Patients and Families

We recruited individuals with recurrent erysipelas infections for which preventive monthly intramuscular benzathine penicillin injections are reimbursed in Finland. We contacted all 960 individuals reimbursed for benzathine penicillin through the National Health Insurance Institution in the year 2000. Of these, 50% (483) gave consent to participate and 25% had a first-degree relative with a history of erysipelas. We then collected blood samples from 204 recurrent erysipelas patients and 124 relatives from 52 pedigrees with two or more family members suffering from erysipelas. The diagnosis of erysipelas was verified from hospital records for all patients except for six who self-reported to have had erysipelas but no hospital records were available for verification.

An acute erysipelas cohort of 90 patients with acute erysipelas and 90 population controls matched for age and sex was also recruited. An infectious disease specialist recruited the patients from Tampere University Hospital and Hatanpää City Hospital, Tampere, Finland when they were hospitalized for erysipelas. The cohort is described in detail elsewhere [5].

### Genomic Screen for Non-parametric Linkage

**Figure 1:**
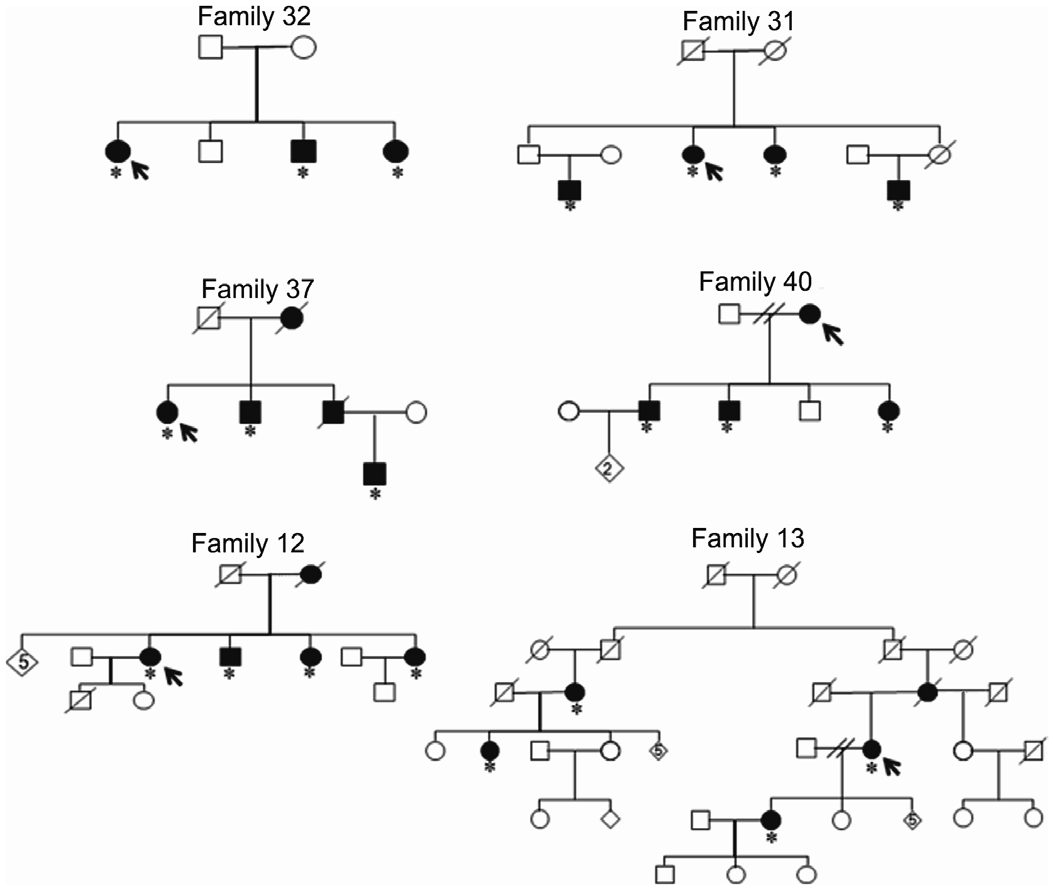
The six most representative families used for initial linkage analysis. Arrows indicate probands and asterisks other family members studied.

Samples from twenty affected individuals from six most representative families (Figure 1) were genotyped using Affymetrix GeneChip Human Mapping 10K Array v 1.0 (Affymetrix, Santa Clara, CA, USA). A total of 11,145 autosomal single nucleotide polymorphisms (SNPs) were used for analysis, with 82–962 SNPs per chromosome. The median physical distance between SNPs was 210 kb (genetic distance ranging from 0.24–1.12 cM), and the average heterozygosity was 0.37. Physical coordinates were mapped against the GRCh37.2 human genome assembly and the deCODE genetic map was used for genetic locations [22].

MERLIN (Multipoint Engine for Rapid Likelihood Inference) software [23] was used for multipoint nonparametric linkage (NPL) analysis. Allele frequencies were estimated from data on 20 affected individuals, and SNPs with unlikely genotypes were removed prior to analysis. The genome-wide significance of NPL_all_ scores was estimated by simulating data 100 times with MERLIN and extracting the highest NPL_all_ score from each simulation. The minimum NPL_all_ score for suggestive linkage was 2.1 (occurring once at random in a genome scan) and the threshold for significant linkage 4.77 (occurring with a 5% probability in a genome scan). Non-parametric linkage analysis was repeated using Caucasian allele frequency estimates obtained from Affymetrix.

### Verification of Linkage Peaks

The NPL results were verified with 31 microsatellites surrounding the suggested linkage peaks at 3q22-24, 9q34, 21q22, and 22q13 at approximately 2 cM (0.85–2.2 Mb) intervals (Table 1). We genotyped 91 individuals (54 affected, 31 non-affected, and six who were defined unconfirmed as their erysipelas diagnosis could not be verified) from 19 families using PCR followed by capillary electrophoresis. Further fine mapping of the 9q34 linkage peak (131527468 - 135831155 bp) was performed with 22 microsatellites from 130457260 to 136035489 bp (Table 2). PCR assays were performed in 5 µl volumes containing 20 ng of DNA with standard reagent concentrations and temperature profiles. Fluorescently labeled PCR products were run on an ABI 377 sequencer. Allele calling was performed using Genotyper 2.0 (Applied Biosystems).

**Table 1:** Non-parametric linkage results from using additional microsatellite markers surrounding the suggested linkage peaks.

Chromosomal locus	Marker	Genetic locus (cM)	Physical locus (bp)	Configuration 0	Configuration 0	Configuration 2	Mb between markers (total area on Chr)
				NPL_all_	Genome-wide p-value	NPL_all_	
3q22-24	D3S1238	142.60	133915404	0.98	0.692	1.10	
	D3S1576	144.46	137420009	1.04	0.660	1.16	3.50
	D3S1309	146.63	140726546	0.96	0.703	1.05	3.31
	D3S3694	148.82	142190878	0.95	0.708	1.04	1.46
	D3S1569	150.58	143371594	0.85	0.762	0.94	1.18
	D3S1593	152.31	145328023	0.86	0.761	0.96	1.96
	D3S1306	154.18	147801038	1.06	0.651	1.04	2.47
	D3S1555	156.59	148807201	1.11	0.629	0.94	1.01
	D3S1299	158.56	150183860	1.02	0.669	0.72	1.38
	D3S1279	160.19	151025260	0.91	0.732	0.61	0.84
	D3S3531	162.79	154213366	0.75	0.803	0.46	3.19
	D3S1607	164.61	156964125	0.43	0.921	0.14	2.75
	D3S3579	166.45	160580277	0.29	0.959	0.00	3.62 (26.66)
9q34	D9S290	136.40	131527468	2.31	0.074	2.26	
	**D9S159**	**138.13**	**132369694**	**2.77**	**0.026**	**2.71**	**0.84**
	D9S1863	140.35	133499845	2.67	0.034	2.62	1.08
	D9S313	141.04	133887414	2.66	0.035	2.60	0.39
	D9S179	143.19	135091628	2.42	0.063	2.36	1.20
	D9S1199	144.51	135831155	2.18	0.104	2.14	0.74 (4.25)
21q22	D21S262	35.68	33816306	1.20	0.560	1.34	
	D21S1898	37.68	34609231	1.32	0.491	1.47	0.79
	D21S1445	38.48	35375662	1.33	0.482	1.48	0.77
	D21S1920	38.82	35697192	1.31	0.497	1.46	0.32
	D21S1895	39.51	36351059	0.92	0.723	1.01	0.65 (2.53)
22q13	D22S1171	53.45	44417584	0.44	0.914	0.49	
	D22S1159	54.62	44750615	0.39	0.933	0.48	0.33
	D22S274	56.47	45269152	0.51	0.889	0.66	0.52
	D22S1141	58.59	45718900	0.37	0.938	0.52	0.45
	D22S1153	61.82	46404511	0.84	0.766	0.98	0.69
	D22S1161	67.43	47596331	0.29	0.959	0.38	1.19
	D22S1170	70.98	48350736	0.22	0.967	0.29	0.75 (3.93)

The most significant locus is highlighted in bold. Physical coordinates were mapped against the GRCh37.2 human genome assembly. The deCODE genetic map was used for genetic locations [22] and for markers absent on the deCODE map, genetic coordinates were estimated with linear interpolation using the markers’ physical coordinates. cM =  centiMorgan.

NPL_all_ = non-parametric linkage score when testing for allele sharing among affected individuals.

**Table 2:** Finemapping of the 9q34 linkage peak region with microsatellite markers.

Marker	Physicallocus (bp)	Candidategenes	Mouse GASgenes^a^
	130026756–130155828		*Garnl3* ↓
	130882972–130890712		*Ptges2* ↑
D9S918	130457260		
	130500596–130541048		*Sh2d3c* ↑
D9S1827	131001749		
D9S290*	131527468		
	131873228–131911225		*Ppp2r4* ↓
D9S752*	131951047		
D9S972*	132051085		
D9S65*	**132190620**		
D9S115*	132248174		
D9S1795*	132306492		
D9S159*	132369694		
D9S1831*	132421728		
	132427920–132484953	*PRRX2*	
D9S62*	132461670		
	132500615–132515344	*PTGES*	*Ptges* ↑
D9S1861*	133370746		
D9S118*	133419164		
D9S1863*	133499845		
	133589268–133763062	*ABL1*	
	133777825–133814455	*FIBCD1*	
	133884504–133968446	*LAMC3*	
D9S313*	133887414		
D9S903*	133935886		
D9S64*	**134380110**		
D9S179*	135091628		
D9S1847*	135436949		
D9S1830*	135715761		
D9S1199*	135831155		
D9S2157	136035489		
	139743256–139745490		*Phpt1* ↓
	139756571–139760738		*Edf1* ↓
	139942553–139948505		*Entpd2* ↓
	140069236–140083057		*Anapc2* ↑

The linkage area is marked by asterisks and the highest linkage peaks are highlighted in bold.

a Genes in the mouse quantitative trait locus for susceptibility to group A streptococcal (GAS) infections on chromosome 2 [18]. (↑) Genes up regulated and, (↓) down regulated in GAS susceptible mouse strains.

MERLIN was used for multipoint NPL analysis as described above. Allele frequencies were estimated from all individuals, non-affected individuals were assigned affection status unknown, and Mendelian inconsistent genotypes were removed prior to analysis. Linkage analysis was done using two configurations: 0; the unconfirmed affected individuals were analyzed as unknown, and 2; they were analyzed as affected. The genome-wide significance of NPL_all_ scores for configuration 0 was estimated by simulating data 1000 times with MERLIN and extracting the highest NPL_all_ score from each simulation. Minimum NPL_all_ score for significant linkage was 2.49, p = 0.05. For fine mapping of 9q34, linkage analysis was done using four configurations: 0; unconfirmed affected individuals were analyzed as unknown, and 2; they were analyzed as affected. In configurations 0_186 and 2_186, analysis was identical except that allele 186 was called for marker D9S65.

### Follow-up Genomic Screening with Higher-density Array

We selected 15 affected patients and 15 unaffected controls for additional genomic screening with the Affymetrix GeneChip Human Mapping 250KSty Array to search for possible allele or haplotype associations assuming a strong genetic effect. Twelve patients were from the families 1, 2, 4, 5, 8, 9, 12, 14, 22, 32, 37, and 38, and their genetically independent family members served as controls. Three patients and three controls were from the acute cohort. Genotypes were called with BRLMM using Affymetrix default parameters. Analysis focused on the defined linkage peaks: 3q22-24 (D3S1306-D3S1299), 9q34 (D9S290-D9S1863), 21q22 (D21S1898-D21S1920), and 22q31 (D22S1159-D22S1141) (Table 1). To evaluate potential differences of haplotype frequencies between cases and controls, shared heterozygosity among cases was checked, allelic association was analyzed by using Haploview, and haplotype association was analyzed by both Haploview and Haplotype Pattern Mining [24], [25].

### Candidate Gene Analyses

Altogether five candidate genes in the 9q34 linkage peak were chosen based on their biological relevance in immunity or infections (Table 2). After PCR with flanking intronic primers, all exons were sequenced in index individuals from the six families (11, 12, 13, 28, 40, and 46) showing most significant linkage (Table S1). Similarly, exons and exon-intron boundaries of *AGTR1* (Angiotensin II receptor, type 1) were sequenced in six probands from the families (7, 13, 37, 40, 43, and 46) showing linkage to the 3q22 area. All PCR reactions were performed in 5 µl volumes containing 20 ng of DNA, with standard reagent concentrations and temperature profiles. Sequencing was performed using dye-terminator chemistry and automated sequencers (ABI, Columbia, Maryland, United States). Primer sequences are available on request.

## Results

### Initial Non-parametric Genome-wide Linkage Results

**Figure 2:**
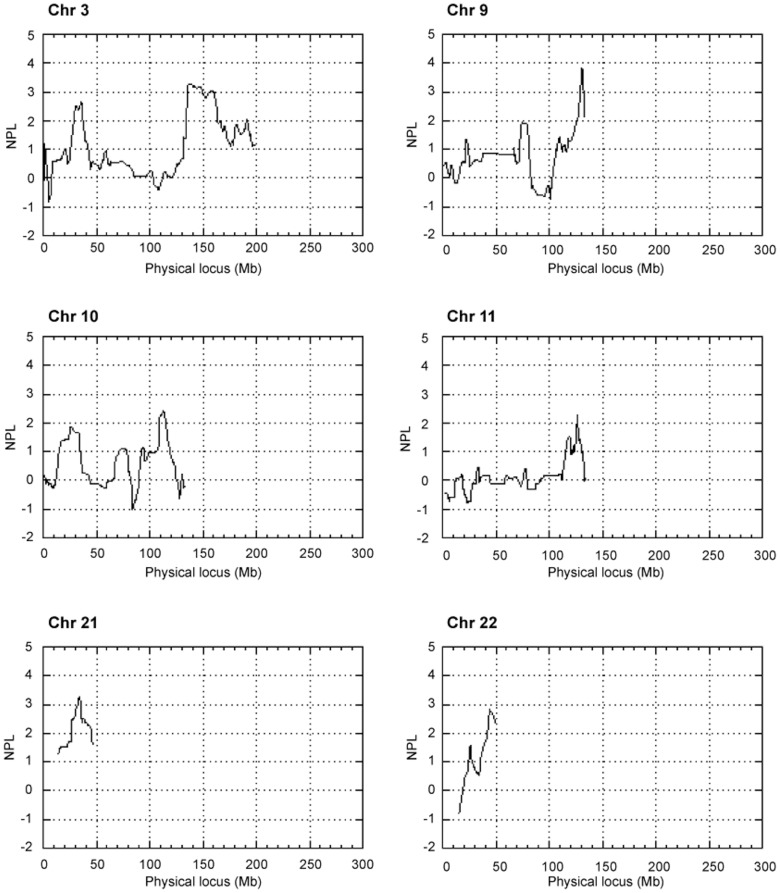
The NPL_all_ scores from initial non-parametric linkage analysis for the chromosomes showing suggestive linkage. Allele frequencies for the Affymetrix HMA10K Array were estimated using 20 affected individuals from six families and MERLIN was used for multipoint NPL analysis.

We found seven suggestive linkage peaks on chromosomes 9q34, 3q22-24, 21q22, 22q13, 3p24, 10q25, and 11q24, in descending order of genomic significance, on the Affymetrix HMA10K Array (Table 3, Figure 2). The strongest linkage was on chromosome 9q34 (rs578802- rs708616), with an NPL_all_ score of 3.84 and a suggestive genome-wide p-value of 0.24. Results were identical when the analysis was repeated with Caucasian allele frequency estimates from Affymetrix, except that the chromosome 3 peak marker moved from 3p24 (rs1994987) to 3p22 (rs2167176) with an NPL_all_ score of 2.64 and a genome-wide P-value 0.94. Generally, different families contributed strongest to the most significant peaks: 9q34 (Families 12 NPL_all_ 3.2 and 13 NPL_all_ 4.8); 3q22-q24 (Families 13 NPL_all_>5 and 37 NPL_all_ 2.0); 21q22 (Families 12 NPL_all_ 3.7 and 21 NPL_all_ 2.5); and 22q13 (Families 12 NPL_all_ 2.0 and 31 NPL_all_ 1.7).

**Table 3:** Non-parametric genome-wide linkage analysis results with MERLIN.

Chromosomal locus	Max NPL_all_	Genome-wide p-value	Marker(s)	Physical locus (bp)
3q22	3.25	0.64	rs361239-rs1429759	136701295–137656598
3p24	2.53	0.97	rs1994987	30456489
3p22	2.64	0.94	rs2167176	35383108
9q34	3.84	0.24	rs578802-rs708616	135453277–135564946
10q25	2.40	0.98	rs1337987-rs959127	113538188–113611569
11q24	2.27	1.00	rs1940007-rs1940006	126754451–126754515
21q22	3.24	0.64	rs743337-rs717205	35265524–35310905
22q13	2.83	0.83	rs719925-rs136578	45758758–45770619

Max NPL_all_ = maximum non-parametric linkage score when testing for allele sharing among affected individuals.

### Verification of Linkage Peaks

Genotyping 91 affected and non-affected individuals from 19 families with 31 microsatellite markers surrounding the four most suggestive linkage peaks on 3q22-24, 9q34, 21q22, and 22q13 revealed one significant linkage peak for 9q34 with the highest NPL_all_ of 2.77 and p = 0.026 for D9S159 (minimum NPL_all_ score for significant linkage was 2.49) (Table 1). Only suggestive linkage was seen for 3q22-24, 21q22, and 22q13 (Table 1). The highest NPL_all_ score was at 9q34 in both configurations. Families 12 and 13 contributed again strongest to the linkage peak on chromosome 9q34, with the highest NPL_all_ scores of 3.64 at D9S179 and 5.02 at D9S313, respectively, and four other families showed suggestive linkage (NPL_all_ scores of 1.34–1.41) (Table S1).

### Chromosome 9q34 Microsatellite Fine Mapping by Microsatellites

The chromosome 9q34 region was further fine mapped with 22 microsatellite markers in the same 91 individuals (Table 2). Highest linkage (NPL_all_ 2.9) was observed at D9S65 (132190620 bp) if allele 186 was called, otherwise it shifted to marker D9S64 (134380110 bp) (NPL_all_ 2.7). NPL plots for the four configurations were essentially unchanged (Table 2, Figure S1).

Altogether, 59 annotated protein-coding genes are located within the chromosome 9q34 linkage peak (D9S290 to D9S1199) (Table 2). The five functionally most interesting genes were sequenced in the index individuals from the six families showing most significant linkage to 9q34 (Table S1, Table 2). *PRRX2* (Paired related homeobox 2) is expressed in proliferating fetal fibroblasts and the developing dermal layer, with lower expression in adult skin. An increase in expression of this gene during fetal but not adult wound healing suggests a role in controlling mammalian dermal regeneration and prevention of scar formation [26]. The *LAMC3* (Laminin, gamma-3) gene belongs to the family of laminins, which are extracellular matrix glycoproteins and the major noncollagenous constituent of basement membranes. They have been implicated in a wide variety of biological processes including intracellular invasion by several bacterial pathogens such as GAS strains [27]. *FIBCD1* (Fibrinogen C domain-containing 1) is a transmembrane endocytic receptor that binds acetylated structures via a highly conserved fibrinogen-related domain (FReD). Ficolins also have FReDs and they play an important role in innate immunity [28]. FIBCD1 binds chitin and has been suggested to control the exposure of intestine to chitin and its fragments, which is important in the immune defense against parasites and fungi and the modulation of immune response [29]. In addition, fibrinogen is a plasma protein that streptococci adhere to in order to avoid host defense. *ABL1* (c-abl oncogene 1, non-receptor tyrosine kinase) is a proto-oncogene which encodes a cytoplasmic and nuclear protein tyrosine kinase implicated in the processes of cell differentiation, cell division, cell adhesion, and stress response. ABL tyrosine kinases are related to the cell penetration of Shigellae and their signaling is required T-cell development and mature T-cell function [30], [31]. Sequencing revealed no specific genetic variations that would implicate any of these genes in erysipelas susceptibility.

*PTGES* (prostaglandin E synthase) is induced by proinflammatory cytokine interleukin 1 beta (IL1B) and synthesizes prostaglandin E2 (PGE2), a key regulator of inflammation by modulating the regulation and activity of T cells and the development and activity of B cells, and by enhancing the production of cytokines and antibodies [32]. PGE2 also modulates the severity of infection caused by GAS [33]. Upon contact with GAS, skin keratinocytes exert a strong proinflammatory response, resulting in the increased expression of several cytokines and the rapid release of PGE2 [34]. *PTGES* is associated with inflammatory diseases, fever, and pain associated with inflammation, and the deletion of *Ptges* leads to an impaired febrile response in mice [35]. We sequenced the introns and 10kb upstream of the transcription start site of *PTGES* as well as the coding region, but found no specific variants, mutations or indels implicating it directly in erysipelas susceptibility.

### Follow-up Genotyping with Higher-density Array

We screened 15 affected patients and 15 unaffected control individuals with the Affymetrix GeneChip Human Mapping 250KSty Array and focused analysis on the previously identified regions on chromosomes 3q22 (D3S1306 to D3S1299), 9q34 (D9S290 to D9S1863), 21q22 (D21S1898 to D21S1920), and 22q23 (D22S1159 to D22S1141). The 3q22 locus was the most significant with several SNPs in the promoter region of the Angiotensin II type receptor 1 (*AGTR1*) between SNPs rs9862062 (148359724 bp) and rs4681157 (148412408 bp) showing nominal association (Table 4).

**Table 4:** Affymetrix HMA250K results for 3q22.

SNP	Physicallocus (bp)	Gene; position	Haploview	Haploview	Shared heterozygosity	Haplotype pattern mining	Haplotype pattern mining
			Associated allele	p-value		p-value	Score
rs2091023	148265311		G	0.787		0.460	16
							
rs12490567	148274505		G	0.775		0.336	32
							
rs1522940	148315687		G	0.389		0.169	59
rs2687661	148319233		T	0.795		**0.046**	101
rs6803324	148335030		A	0.313	x	**0.013**	142
rs6440561	148358582		C	**0.044**	x	**0.012**	194
rs6440562	148358705				x	**0.010**	218
rs9862062*	148359724		**G**	**0.045**	x	**0.013**	259
rs9811115*	148360046		**A**	**0.045**	x	**0.023**	263
rs275679	148368303		**G**	0.129	x	**0.040**	231
rs10513336	148368387				x	0.078	188
rs275711	148374631		**T**	0.230	x	0.084	163
rs718424	148380543		**C**	**0.045**	x	0.082	164
rs2087737	148381522		**T**	**0.045**	x	0.092	140
rs16860674	148382002				x	0.108	90
rs872212	148386747		**T**	0.166	x	0.108	55
rs2012052	148386856		**A**	0.166	x	0.113	36
rs454530	148400657		**C**	0.228	x	0.113	25
rs2638359	148406383		**T**	0.228	x	0.086	19
rs2638358	148406537		**A**	0.228	x	0.119	18
rs2638357	148406619		**A**	0.228	x	0.119	22
rs2933251	148406799		**T**	0.228	x	0.096	39
rs409742	148412365		**C**	0.228	x	0.091	59
rs4681157	148412408		**T**	**0.045**	x	0.080	80
rs12721267	148416327	*AGTR1*; intron 1			x	0.082	76
rs12695877	148427034	*AGTR1*; intron 2				0.105	74
rs1492103	148432964	*AGTR1*; intron 2	G	0.129		0.113	68
rs12695918	148456627	*AGTR1*; intron3				0.123	58
rs13097326	148468746		C	0.197		0.126	50

The haplotype that was significantly associated to erysipelas in Haploview is marked with bold letters in the “Associated allele” column. Significant p-values in Haploview or Haplotype pattern mining (HPM) for individual SNPs are also highlighted in bold. SNPs belonging to the associated haplotype and a significant p-value in Haploview, and with a significant p-value in HPM, and that showed shared heterozygosity among cases are marked with an asterisk.

*AGTR1* exons and exon-intron boundaries were sequenced in six probands from the families showing strongest linkage to the 3q22 region. Twelve known SNPs were identified, including rs5186 (also known as 1166 A/C) in the 3′UTR. The A allele of rs5186 has been associated with increased serum levels of high-sensitivity C-reactive protein and inflammation, and the CC genotype is putatively correlated with hypertension [36], [37] (Table S2). Out of six probands, five were homozygous AA, one heterozygous AC, and none had the CC genotype, thus supporting a potential role in inflammation for the A allele. However, no statistically significant difference in allele frequencies was detected for rs5186 between cases and controls in the acute cohort. No other variants that might explain linkage to this region were found in *AGTR1*.

We chose two *AGTR1* promoter area SNPs (rs9862062 and rs718424) that showed association to erysipelas in Haploview analysis, and genotyped them in the family material and in the acute erysipelas cohort by direct sequencing. The reference G-allele of rs9862062 was suggestively associated in the combined family (probands and marry-ins) and acute erysipelas cohort (Fisher’s exact test, two-tailed p-value 0.006) and the reference T-allele of rs718424 showed suggestive association with a p-value of 0.017.

## Discussion

Individual response to potentially fatal pathogens is modulated by both environmental and host genetic factors [8], [9]. Streptococcal infections can vary from localized pharyngitis or erysipelas to potentially fatal necrotizing fasciitis and sepsis. We have used erysipelas/cellulitis, a localized infection of the skin and underlying subcutaneous tissues to identify 52 families with a possibly increased susceptibility to streptococcal infections. This is to our knowledge the largest systematically collected clinical material on familial segregation of recurrent erysipelas. We performed a linkage scan on the six most informative families segregating erysipelas and found evidence for suggestive linkage in seven chromosomal regions, with a maximum NPL score of 3.84 at 9q34. Further fine mapping of the four most significant regions in all of the collected families revealed significant linkage to the chromosome 9q34 region which is syntenic to mouse chromosome 2 (22 to 34 Mb), where a quantitative trait locus (QTL) for GAS susceptibility in mice has been identified [18]. In mouse, 37 candidate genes involved in immune response, cell signalling, cellular assembly and organization, and lipid metabolism were studied for quantitative expression levels pre- and postinfection in strains resistant and susceptible to severe GAS infection. Genes associated with early immune response and upregulated in susceptible strains and downregulated in resistant strains included *Il1a, Il1rn* (both located on 2q14 in humans), *Ptges* (located on 9q34 the linkage peak identified here), and *Ptges2* (located proximal to the 9q34 linkage peak) (Table 2). Increased production of prostaglandins has also been associated with Gram positive infections including *Streptococcus suis*, group B streptococcal, and GAS skin infections [34], [38]–[40]. However, sequencing of *PTGES* and four other chosen candidate genes in the 9q34 linkage region did not reveal significant genetic variations implicating any of these genes in erysipelas susceptibility. However, it is possible that quantitative expression level analysis of candidate genes could have revealed variation associated with erysipelas [18]. Expression analysis for the candidate genes was not performed in this study. The genes for sequencing were chosen based on their known function and thus, we could have missed genes with yet unknown roles in immunity and infection. The inherent property of genetic linkage is the relatively broad genomic area that is implicated. In this case, the 9q34 region contained 59 genes that in this study were impractical to sequence. Our rationale for choosing target genes was then necessarily based on known functional information and biological plausibility, and we admit this approach has its limitations. More candidate genes will need to be considered as data accumulate. Susceptibility to infection is a complex trait where multiple genes in an immunological pathway or multiple intertwining pathways play a role in disease outcome [18].

Higher density analysis with the Affymetrix HMA250K Array revealed the nominal association to erysipelas of several SNPs in the promoter region of *AGTR1* on 3q22. *AGTR1* is a G-protein-coupled receptor that mediates the major cardiovascular effects of angiotensin II, a potent vasopressor hormone involved in the development of hypertension, atherosclerosis, and insulin resistance. Angiotensin II is the end product of the renin-angiotensin system (RAS), where renin stimulates the production of angiotensin I from angiotensinogen, which is then converted to angiotensin II by angiotensin converting enzyme (ACE). The activation of the RAS correlates with organ injury and mortality in clinical sepsis, possibly by contributing to the enhanced microvascular tone [41]. Angiotensin II also exerts proinflammatory effects on leukocytes, endothelial cells, and vascular smooth muscle cells and by acting through *AGTR1,* it increases the expression of cytokines, chemokines, growth factors, and adhesion molecules [42].

Polymorphisms in both *ACE* and other angiotensinogen genes have been associated with susceptibility to inflammatory diseases such as SLE and psoriasis with frequent tonsillitis [43], [44]. The Angiotensin II pathway also plays a potential role in septic shock [45]. Selected SNPs in *ACE, AGTR1*, and Angiotensin receptor associated protein (*AGTRAP)* showed significant association with increased 28-day mortality to septic shock for the GG genotype of *AGTRAP* rs11121816. AGTRAP interacts specifically with the C-terminal tail of AGTR1, and negatively regulates the receptor leading to a functional desensitization to angiotensin II. Pharmacological blockage of the RAS is used to treat hypertension, diabetic nephropathy, and congestive heart failure, but antigen II receptor blockers (ARBs) and ACE inhibitors also suppress proinflammatory cytokines and reduce oxidative stress. Prior usage of ARBs has been shown to reduce mortality in patients hospitalized for sepsis [46].

Polymorphisms in *AGTR1* and especially the C allele of rs5186 (+1166A>C) have been associated with hypertension and the A allele of rs5186 has been associated with higher serum levels of high-sensitivity C-reactive protein (CRP) and inflammation [36], [37]. Out of our six probands five were homozygous AA, one heterozygous AC, and none had the CC genotype. In the presence of AA or AC genotypes microRNA-155 (miR-155) represses expression of the AGTR1 protein [47]. MiR-155 mediated translational repression can be regulated by, e.g., TGFB1, and MiR-155 expression is significantly increased with the AA or AC genotypes as compared to the CC genotype. MiR-155 is critically involved in the control of specific differentiation processes in the immune response. It functions specifically in regulating T helper cell differentiation and the germinal center reaction to produce an optimal T cell–dependent antibody response, mediated at least partly by regulating cytokine production [48]. Furthermore, the loss of MiR-155 leads to an overall attenuation of immune responses in mouse [49]. High CRP levels and leukocyte counts (i.e., a more severe inflammatory response) in erysipelas are associated with recurrence of erysipelas [5]. Our finding of predominance of the A-allele in our six probands is consistent with these earlier observations. Interestingly, *AGTR1* and *PTGES* are involved in the same pathway, as *AGTR1* induces the production of COX, which coverts arachidonic acid into Prostaglandin H2 that in turn is converted by *PTGES* into Prostaglandin E2.

We found evidence for host genetic factors influencing susceptibility to bacterial non-necrotizing erysipelas/cellulitis, but did not find a common susceptibility factor in all families. We did not find linkage or association with the HLA region previously linked with GAS infection severity in humans [19], [20]. It is likely that as the inflammatory pathways are very complex and the defense against infections is under strong selection, different families are likely to have individual genetic susceptibilities. Genetic heterogeneity makes it difficult to find significant correlations, which is a common pitfall of studies on host genetic factors predisposing to infections. Much larger patient and control groups will be needed to verify these preliminary results. However, our linkage peak and the region of strongest association coincide with genes and pathways suggested to play important roles in susceptibility to streptococcal infections. The identification of the susceptibility genes would help to understand better the course of infections and ultimately reduce morbidity.

## Supporting Information

Figure S1 **NPL plots for the fine mapping of the chromosome 9q34 linkage peak with 22 microsatellite markers.** The NPL plots for the four configurations were essentially identical. MERLIN was used for multipoint NPL analyses using four configurations. (*A*) In configuration 0, unconfirmed affected individuals were analyzed as unknown, and (*B*) in configuration 2, they were analyzed as affected. In configurations (*C*) 0_186 and (*D*) 2_186, analysis was identical to configurations 0 and 2, respectively, except that allele 186 was called for marker D9S65.
(TIF)

Table S1 **Family-wise NPL_all_ scores for the 9q34 linkage region.** Families showing significant linkage are shaded dark grey. Families showing suggestive linkage are shaded light grey.
(DOCX)

Table S2 **SNPs found in the family probands in** ***AGTR1*** **.**
(DOCX)

## References

[1] Bisno AL, Stevens DL (1996) Streptococcal infections of skin and soft tissues. N Engl J Med 334: 240–245. 8532002 10.1056/NEJM199601253340407

[2] Siljander T, Karppelin M, Vähäkuopus S, Syrjänen J, Toropainen M, et al (2008) Acute bacterial, nonnecrotizing cellulitis in Finland: microbiological findings. Clin Infect Dis 46: 855–861. 18260753 10.1086/527388

[3] Pavlotsky F, Amrani S, Trau H (2004) Recurrent erysipelas: risk factors. J Dtsch Dermatol Ges 2: 89–95. 16279242 10.1046/j.1439-0353.2004.03028.x

[4] Bjornsdottir S, Gottfredsson M, Thorisdottir AS, Gunnarsson GB, Rikardsdottir H, et al (2005) Risk factors for acute cellulitis of the lower limb: a prospective case-control study. Clin Infect Dis 41: 1416–1422. 16231251 10.1086/497127

[5] Karppelin M, Siljander T, Vuopio-Varkila J, Kere J, Huhtala H, et al (2010) Factors predisposing to acute and recurrent bacterial non-necrotizing cellulitis in hospitalized patients: a prospective case-control study. Clin Microbiol Infect 16: 729–734. 19694769 10.1111/j.1469-0691.2009.02906.x

[6] McNamara DR, Tleyjeh IM, Berbari EF, Lahr BD, Martinez J, et al (2007) A predictive model of recurrent lower extremity cellulitis in a population-based cohort. Arch Intern Med 167: 709–715. 17420430 10.1001/archinte.167.7.709

[7] Chatellier S, Ihendyane N, Kansal RG, Khambaty F, Basma H, et al (2000) Genetic relatedness and superantigen expression in group A streptococcus serotype M1 isolates from patients with severe and nonsevere invasive diseases. Infect Immun 68: 3523–3534. 10816507 10.1128/iai.68.6.3523-3534.2000 PMC97638

[8] Burgner D, Jamieson SE, Blackwell JM (2006) Genetic susceptibility to infectious diseases: big is beautiful, but will bigger be even better? Lancet Infect Dis 6: 653–663. 17008174 10.1016/S1473-3099(06)70601-6 PMC2330096

[9] Casanova J-L (2007) Abel (2007) Human genetics of infectious diseases: a unified theory. EMBO J 26: 915–922. 17255931 10.1038/sj.emboj.7601558 PMC1852849

[10] Vannberg FO, Chapman SJ, Hill AV (2011) Human genetic susceptibility to intracellular pathogens. Immunol Rev 240: 105–116. 21349089 10.1111/j.1600-065X.2010.00996.x

[11] Bellamy R, Beyers N, McAdam KP, Ruwende C, Gie R, et al (2000) Genetic susceptibility to tuberculosis in Africans: a genome-wide scan. Proc Natl Acad Sci U S A 97: 8005–8009. 10859364 10.1073/pnas.140201897 PMC16660

[12] Siddiqui MR, Meisner S, Tosh K, Balakrishnan K, Ghei S, et al (2001) A major susceptibility locus for leprosy in India maps to chromosome 10p13. Nat Genet 27: 439–441. 11279529 10.1038/86958

[13] Bucheton B, Abel L, El-Safi S, Kheir MM, Pavek S, et al (2003) A major susceptibility locus on chromosome 22q12 plays a critical role in the control of kala-azar. Am J Hum Genet 73: 1052–1060. 14557985 10.1086/379084 PMC1180485

[14] Mira MT, Alcaïs A, Van Thuc N, Thai VH, Huong NT, et al (2003) Chromosome 6q25 is linked to susceptibility to leprosy in a Vietnamese population. Nat Genet 33: 412–415. 12577057 10.1038/ng1096

[15] Aziz RK, Kansal R, Abdeltawab NF, Rowe SL, Su Y, et al (2007) Susceptibility to severe Streptococcal sepsis: use of a large set of isogenic mouse lines to study genetic and environmental factors. Genes Immun 8: 404–415. 17525705 10.1038/sj.gene.6364402

[16] Medina E, Lengeling A (2005) Genetic regulation of host responses to group A streptococcus in mice. Brief Funct Genomic Proteomic 4: 248–257. 16420750 10.1093/bfgp/4.3.248

[17] Goldmann O, Lengeling A, Böse J, Bloecker H, Geffers R, et al (2005) The role of the MHC on resistance to group A streptococci in mice. J Immunol 175: 3862–3872. 16148132 10.4049/jimmunol.175.6.3862

[18] Abdeltawab NF, Aziz RK, Kansal R, Rowe SL, Su Y, et al (2008) An unbiased systems genetics approach to mapping genetic loci modulating susceptibility to severe streptococcal sepsis. PLoS Pathog 4: e1000042. 18421376 10.1371/journal.ppat.1000042 PMC2277464

[19] Kotb M, Norrby-Teglund A, McGeer A, El-Sherbini H, Dorak MT, et al (2002) An immunogenetic and molecular basis for differences in outcomes of invasive group A streptococcal infections. Nat Med 12: 1398–1404. 10.1038/nm1202-800 12436116

[20] Nooh MM, Nookala S, Kansal R, Kotb M (2011) Individual genetic variations directly effect polarization of cytokine responses to superantigens associated with streptococcal sepsis: implications for customized patient care. J Immunol 186: 3156–3163. 21282506 10.4049/jimmunol.1002057

[21] Blackwell JM, Jamieson SE, Burgner D (2009) HLA and infectious diseases. Clin Microb Rev 22: 370–385. 10.1128/CMR.00048-08 PMC2668228 19366919

[22] Kong A, Gudbjartsson DF, Sainz J, Jonsdottir GM, Gudjonsson SA, et al (2002) A high-resolution recombination map of the human genome. Nat Genet 31: 241–247. 12053178 10.1038/ng917

[23] Abecasis GR, Cherny SS, Cookson WO, Cardon LR (2002) Merlin–rapid analysis of dense genetic maps using sparse gene flow trees. Nat Genet 30: 97–101. 11731797 10.1038/ng786

[24] Barrett JC, Fry B, Maller J, Daly MJ (2005) Haploview: Analysis and visualisation of LD and haplotype maps. Bioinformatics 21: 263–265. 15297300 10.1093/bioinformatics/bth457

[25] Toivonen HTT, Onkamo P, Vasko K, Ollikainen V, Sevon P, et al.. (2000) Gene mapping by haplotype pattern mining. In: Proceedings of 1st IEEE International Symposium on Bioinformatics and Biomedical Engineering. 99–108.

[26] Stelnicki E, Arbeit J, Cass DL, Saner C, Harrison M, et al (1998) Modulation of the human homeobox genes PRX-2 and HOXB13 in scarless fetal wounds. J Invest Dermatol 111: 57–63. 9665387 10.1046/j.1523-1747.1998.00238.x

[27] Cue D, Dombek PE, Lam H, Cleary PP (1998) Streptococcus pyogenes serotype M1 encodes multiple pathways for entry into human epithelial cells. Infect Immun 66: 4593–4601. 9746555 10.1128/iai.66.10.4593-4601.1998 PMC108566

[28] Thomsen T, Schlosser A, Holmskov U, Sorensen GL (2011) Ficolins and FIBCD1: soluble and membrane bound pattern recognition molecules with acetyl group selectivity. Mol Immunol 48: 369–381. 21071088 10.1016/j.molimm.2010.09.019

[29] Schlosser A, Thomsen T, Moeller JB, Nielsen O, Tornøe I, et al (2009) Characterization of FIBCD1 as an acetyl group-binding receptor that binds chitin. J Immunol 183: 3800–3809. 19710473 10.4049/jimmunol.0901526

[30] Burton EA, Pendergast AM, Aballay A (2006) The Caenorhabditis elegans ABL-1 tyrosine kinase is required for Shigella flexneri pathogenesis. Appl Environ Microbiol 72: 5043–5051. 16820504 10.1128/AEM.00558-06 PMC1489310

[31] Gu JJ, Ryu JR, Pendergast AM (2009) Abl tyrosine kinases in T-cell signaling. Immunol Rev 228: 170–183. 19290927 10.1111/j.1600-065X.2008.00751.x PMC2678244

[32] Harris SG, Padilla J, Koumas L, Ray D, Phipps RP (2002) Prostaglandins as modulators of immunity. Trends Immunol 23: 1144–150. 10.1016/s1471-4906(01)02154-8 11864843

[33] Goldmann O, Hertzén E, Hecht A, Schmidt H, Lehne S, et al (2010) Inducible cyclooxygenase released prostaglandin E2 modulates the severity of infection caused by Streptococcus pyogenes. J Immunol 185: 2372–2381. 20644176 10.4049/jimmunol.1000838

[34] Wang B, Ruiz N, Pentland AP, Caparon M (1997) Keratinocyte proinflammatory responses to adherent and nonadherent group A streptococci. Infect Immun 65: 2119–2126. 9169741 10.1128/iai.65.6.2119-2126.1997 PMC175293

[35] Saha S, Engström L, Mackerlova L, Jakobsson PJ, Blomqvist A (2005) Impaired febrile responses to immune challenge in mice deficient in microsomal prostaglandin E synthase-1. Am J Physiol Regul Integr Comp Physiol 288: R1100–1107. 15677520 10.1152/ajpregu.00872.2004

[36] Suchankova P, Henningsson S, Olsson M, Baghaei F, Rosmond R, et al (2009) Association between the AGTR1 polymorphism +1166A>C and serum levels of high-sensitivity C-reactive protein. Regul Pept 152: 28–32. 19026696 10.1016/j.regpep.2008.11.001

[37] Palatini P, Ceolotto G, Dorigatti F, Mos L, Santonastaso M, et al (2009) Angiotensin II type 1 receptor gene polymorphism predicts development of hypertension and metabolic syndrome. Am J Hypertens 22: 208–214. 19023273 10.1038/ajh.2008.319

[38] Ruiz N, Wang B, Pentland A, Caparon M (1998) Streptolysin O and adherence synergistically modulate proinflammatory responses of keratinocytes to group A streptococci. Mol Microbiol 27: 337–346. 9484889 10.1046/j.1365-2958.1998.00681.x

[39] Maloney CG, Thompson SD, Hill HR, Bohnsack JF, McIntyre TM, et al (2000) Induction of cyclooxygenase-2 by human monocytes exposed to group B streptococci. J Leukoc Biol 67: 615–621. 10811000 10.1002/jlb.67.5.615

[40] Jobin MC, Gottschalk M, Grenier D (2006) Upregulation of prostaglandin E2 and matrix metalloproteinase 9 production by human macrophage-like cells: synergistic effect of capsular material and cell wall from Streptococcus suis. Microb Pathog 40: 29–34. 16324819 10.1016/j.micpath.2005.10.003

[41] Doerschug K, Delsing AS, Schmidt GA, Ashare A (2010) Renin-angiotensin system activation correlates with microvascular dysfunction in a prospective cohort study of clinical sepsis. Critical Care 14: R24. 20175923 10.1186/cc8887 PMC2875539

[42] Mackenzie A (2011) Endothelium-derived vasoactive agents, AT1 receptors and inflammation. Pharmacol Ther 131: 187–203. 21115037 10.1016/j.pharmthera.2010.11.001

[43] Sato H, Akai Y, Iwano M, Kurumatani N, Kurioka H, et al (1998) Association of an insertion polymorphism of angiotensin-converting enzyme gene with the activity of systemic lupus erythematosus. Lupus 7: 530–534. 9863894 10.1191/096120398678920622

[44] Vasku V, Vasku A, Izakovicova Holla L, Tsoplova S, Kankova K, et al (2000) Polymorphisms in inflammation genes (angiotensinogen, TAP1 and TNF-beta) in psoriasis. Arch Dermatol Res 292: 531–534. 11194890 10.1007/s004030000176

[45] Nakada TA, Russell JA, Boyd JH, McLaughlin L, Nakada E, et al (2011) Association of angiotensin II type 1 receptor-associated protein gene polymorphism with increased mortality in septic shock. Crit Care Med 39: 1641–1648. 21423001 10.1097/CCM.0b013e318218665a

[46] Mortensen EM, Restrepo MI, Copeland LA, Pugh JA, Anzueto A, et al (2008) Impact of previous statin and angiotensin II reseptor blocker use on mortality in patients hospitalized with sepsis. Pharmacotherapy 27: 1619–1626. 10.1592/phco.27.12.1619 18041882

[47] Martin MM, Lee EJ, Buckenberger JA, Schmittgen TD, Elton TS (2006) MicroRNA-155 regulates human angiotensin II type 1 receptor expression in fibroblasts. J Biol Chem 281: 18277–18284. 16675453 10.1074/jbc.M601496200

[48] Thai TH, Calado DP, Casola S, Ansel KM, Xiao C, et al (2007) Regulation of the germinal center response by microRNA-155. Science 316: 604–608. 17463289 10.1126/science.1141229

[49] Rodriguez A, Vigorito E, Clare S, Warren MV, Couttet P, et al (2007) Requirement of bic/microRNA-155 for normal immune function. Science 316: 608–611. 17463290 10.1126/science.1139253 PMC2610435

