# Supplementary figures and images for: Genetic Susceptibility to Non-Necrotizing Erysipelas/Cellulitis
Source: PLoS One. 2013 Feb 20;8(2):e56225. doi: 10.1371/journal.pone.0056225 (PMC3577772; doi:10.1371/journal.pone.0056225)

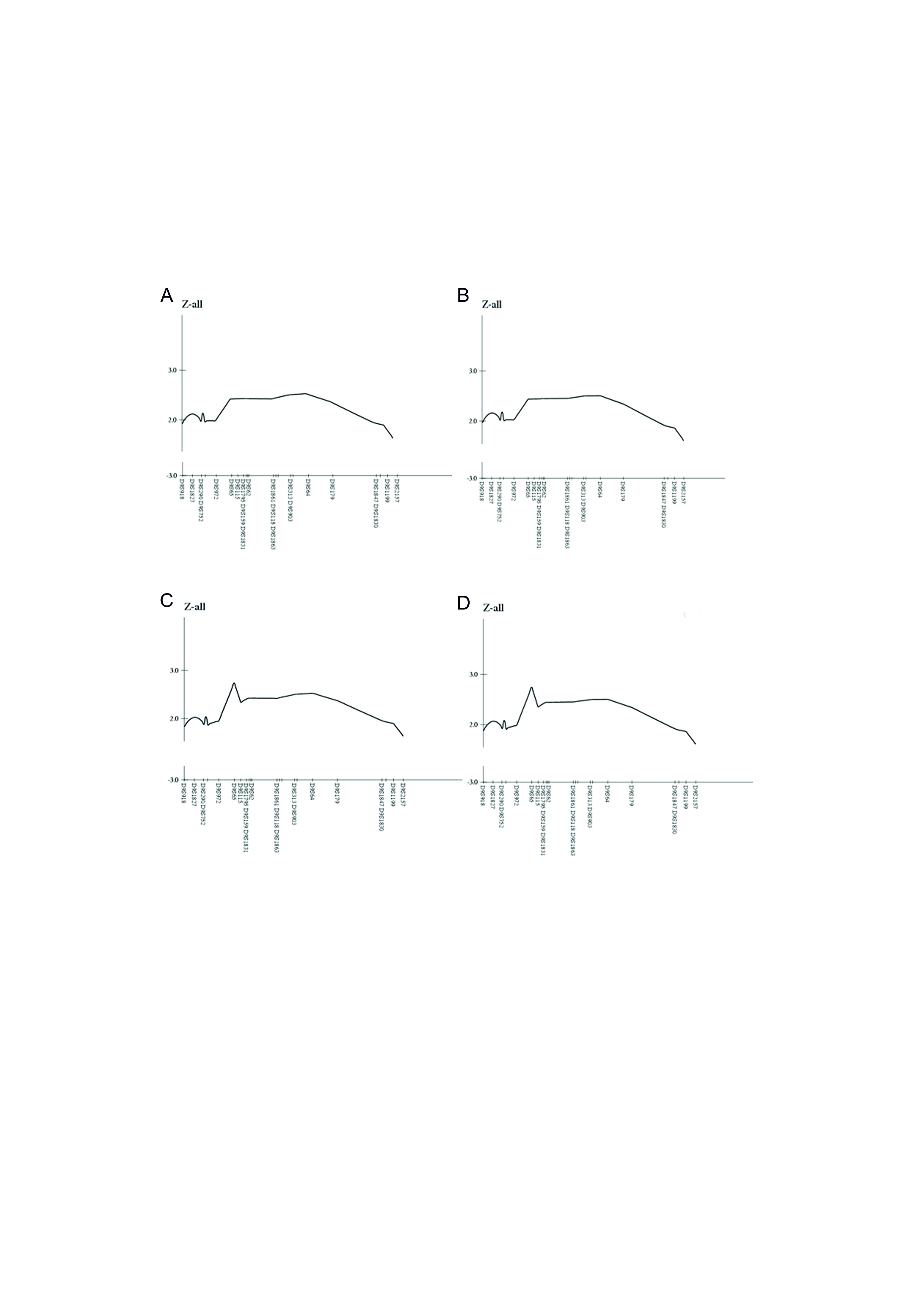

Supplement: Figure S1 — NPL plots for the fine mapping of the chromosome 9q34 linkage peak with 22 microsatellite markers. The NPL plots for the four configurations were essentially identical. MERLIN was used for multipoint NPL analyses using four configurations. (A) In configuration 0, unconfirmed affected individuals were analyzed as unknown, and (B) in configuration 2, they were analyzed as affected. In configurations (C) 0_186 and (D) 2_186, analysis was identical to configurations 0 and 2, respectively, except that allele 186 was called for marker D9S65. (TIF) [file pone.0056225.s001.tif]
