# Supplementary material for: Genetic Susceptibility to Non-Necrotizing Erysipelas/Cellulitis
Source: PLoS One. 2013 Feb 20;8(2):e56225. doi: 10.1371/journal.pone.0056225 (PMC3577772; doi:10.1371/journal.pone.0056225)
Supplement: Table S1 — Family-wise NPLall scores for the 9q34 linkage region. Families showing significant linkage are shaded dark grey. Families showing suggestive linkage are shaded light grey. (DOCX) [file pone.0056225.s002.docx]

Table S1. Family-wise NPL_all_ scores for the 9q34 linkage region. Families showing significant linkage are shaded dark grey. Families showing suggestive linkage are shaded light grey.

| **Marker** | | D9S290 | D9S159 | D9S1863 | D9S313 | D9S179 | D9S1199 |
| --- | --- | --- | --- | --- | --- | --- | --- |
| **Physical locus (bp)** | | 131527468 | 132369694 | 133499845 | 133887414 | 135091628 | 135831155 |
| **Family** | **13** | 4.776 | 4.959 | 5.021 | 5.022 | 4.868 | 4.659 |
|  | **12** | 1.682 | 3.613 | 3.640 | 3.641 | 3.643 | 3.604 |
|  | **46** | 1.399 | 1.412 | 1.410 | 1.409 | 1.395 | 1.376 |
|  | **11** | 1.386 | 1.407 | 1.410 | 1.409 | 1.397 | 1.379 |
|  | **28** | 1.412 | 1.401 | 1.409 | 1.408 | 1.391 | 1.365 |
|  | **40** | 1.342 | 1.342 | 1.341 | 1.341 | 1.341 | 1.320 |
|  | **31** | 0.816 | 0.816 | 0.816 | 0.817 | 0.816 | 0.816 |
|  | **2** | -0.378 | -0.376 | -0.378 | -0.377 | -0.376 | -0.378 |
|  | **5** | -0.447 | -0.441 | -0.439 | -0.440 | -0.447 | -0.470 |
|  | **6** | 0.049 | 0.000 | -0.002 | -0.002 | 0.074 | 0.110 |
|  | **7** | 0.235 | -0.006 | -0.002 | -0.003 | 0.261 | 0.307 |
|  | **25** | -1.414 | -0.743 | -1.414 | -1.414 | -1.413 | -1.378 |
|  | **30** | -0.025 | -0.012 | -0.034 | -0.036 | -0.044 | 0.035 |
|  | **32** | -0.106 | -0.186 | 0.083 | 0.071 | -0.186 | -0.746 |
|  | **37** | 0.088 | -0.189 | -0.178 | -0.173 | -0.169 | -0.172 |
|  | **38** | 0.315 | 0.356 | 0.338 | 0.322 | -0.500 | -1.010 |
|  | **41** | -1.213 | -1.287 | -1.391 | -1.414 | -1.414 | -1.381 |
|  | **43** | 0.246 | -0.003 | -0.004 | 0.004 | -0.045 | 0.034 |
|  | **45** | -0.078 | 0.014 | 0.014 | -0.007 | -0.047 | 0.032 |
