# Supplementary material for: Genetic Susceptibility to Non-Necrotizing Erysipelas/Cellulitis
Source: PLoS One. 2013 Feb 20;8(2):e56225. doi: 10.1371/journal.pone.0056225 (PMC3577772; doi:10.1371/journal.pone.0056225)
Supplement: Table S2 — SNPs found in the family probands in AGTR1 . (DOCX) [file pone.0056225.s003.docx]

Table S2. SNPs found in the family probands in *AGTR1*.

| **SNP** | **Physical locus (bp)** | **Family** | | | | | | ***AGTR1* location** |
| --- | --- | --- | --- | --- | --- | --- | --- | --- |
|  |  | **7** | **13** | **37** | **40** | **43** | **46** |  |
|  |  | **Genotype** | | | | | |  |
| rs422858 | 148415484 | AA | AC | AA | AA | AA | AA | promotor |
| rs387967 | 148415485 | GG | GC | GG | GG | GG | GG | promotor |
| rs275653 | 148415545 | AA | AG | AA | AA | AA | AA | promotor |
| rs2276736 | 148425873 | AA | AG | AG | AA | AG | GG | intron 2 |
| rs2276735 | 148425988 | GG | GA | GA | GG | GA | AA | intron 2 |
| rs388915 | 148447756 | AA | GA | AA | AA | AA | GA | intron 2 |
| rs1800766 | 148457642 | TT | TC | TT | TT | TT | TT | exon 4 (5'UTR) |
| rs5182 | 148459395 | TT | TT | CT | CT | TT | TT | exon 5 (coding synonymous) |
| rs5186 | 148459988 | AA | AA | AA | AC | AA | AA | exon 5 (3'UTR) |
| rs1799870 | 148460037 | CA | CA | CA | CA | CA | CA | exon 5 (3'UTR) |
| rs440881 | 148460467 | AA | AA | AA | AA | AA | AA | exon 5 (3'UTR) |
| rs380400 | 148460700 | AA | AA | GA | AA | AA | AA | exon 5 (3'UTR) |
